# At the Start of the Sarcomere: A Previously Unrecognized Role for Myosin Chaperones and Associated Proteins during Early Myofibrillogenesis

**DOI:** 10.1155/2012/712315

**Published:** 2012-01-30

**Authors:** J. Layne Myhre, David B. Pilgrim

**Affiliations:** Department of Biological Sciences, CW405, Biological Sciences Building, University of Alberta, Edmonton, AB, Canada T6G 2E9

## Abstract

The development of striated muscle in vertebrates requires the assembly of contractile myofibrils, consisting of highly ordered bundles of protein filaments. Myofibril formation occurs by the stepwise addition of complex proteins, a process that is mediated by a variety of molecular chaperones and quality control factors. Most notably, myosin of the thick filament requires specialized chaperone activity during late myofibrillogenesis, including that of Hsp90 and its cofactor, Unc45b. Unc45b has been proposed to act exclusively as an adaptor molecule, stabilizing interactions between Hsp90 and myosin; however, recent discoveries in zebrafish and *C. elegans* suggest the possibility of an earlier role for Unc45b during myofibrillogenesis. This role may involve functional control of nonmuscle myosins during the earliest stages of myogenesis, when premyofibril scaffolds are first formed from dynamic cytoskeletal actin. This paper will outline several lines of evidence that converge to build a model for Unc45b activity during early myofibrillogenesis.

## 1. Introduction

The field of regenerative medicine represents the dawn of a transformative era, as embryonic stem cell treatments move into clinical trials after many years of laboratory research. The first successful surgeries involving complex, stem-cell-derived muscular organs have recently been reported [1, 2], and these treatments are particularly promising for the regeneration of muscle and connective tissue [3, 4]. This is transformative not only because of the potential of stem-cell therapies for treating previously incurable conditions involving damage, loss, or congenital deficiencies of complex organs, but also because it represents the convergence of biomedical tissue engineering and developmental molecular biology [5–7]. As new medical treatments begin to achieve development of whole tissues in adult bodies, rather than the healing of damage to preexisting tissues, developmental biology must provide the insight to guide our understanding of migration, proliferation, and differentiation of stem cells into functional tissues and organs.

The use of regenerative medicine for the treatment of striated muscle diseases, particularly cardiomyopathy, has recently made significant progress [7, 8]. Striated muscle cells, comprising both skeletal muscle and cardiac muscle, contain large, highly-ordered, contractile units of insoluble protein called sarcomeres, arranged in linear arrays of myofibrils. Sarcomeres contain repeating patterns of overlapping cytoskeletal and motor protein filaments, causing the striated appearance of muscle tissue and providing contractile function (reviewed in [9]). Numerous skeletal and cardiac myopathies are associated with mutations in genes involved with sarcomere formation [10–12]. Although the process of cellular differentiation in striated muscle has been well studied, and most components of the sarcomere are likely known, the early events leading to the assembly and patterning of these protein complexes remain poorly understood. Research has focused primarily on the genetic regulation of myogenesis, and on the protein structure of mature sarcomeres, rather than the molecular events guiding the process of myofibril assembly during development. As a result, much is known about the expression of muscle-specific regulatory transcription factors in determined mesenchymal cells, aggregating myoblasts, and fusing myotubes, but little is known about the protein processing and assembly of the sarcomere components and their intermediary structures.

Growing recognition of the role of molecular chaperones in the assembly of large protein complexes makes it clear that the development of striated muscle tissue requires more than merely the sequential expression of sarcomeric genes. Rather, myogenesis must involve highly specific and regulated steps of protein folding and assembly, involving both general and myocyte-specific molecular chaperones, cochaperones, scaffolds, and intermediate structures. This is coupled with dynamic turnover of proteins through proteasome-mediated degradation, resulting in a system of development and repair that allows complex assembly without protein aggregation. Over the past two decades, several models have been proposed of the earliest stages of myofibrillogenesis, focused on the stepwise nucleation and incorporation of sarcomere components, beginning with stress-fiber-like structures in differentiating myoblasts.

## 2. Current Models of Vertebrate Sarcomere Formation

### 2.1. Overview of the Cellular Events of Myogenesis

**Figure 1:**
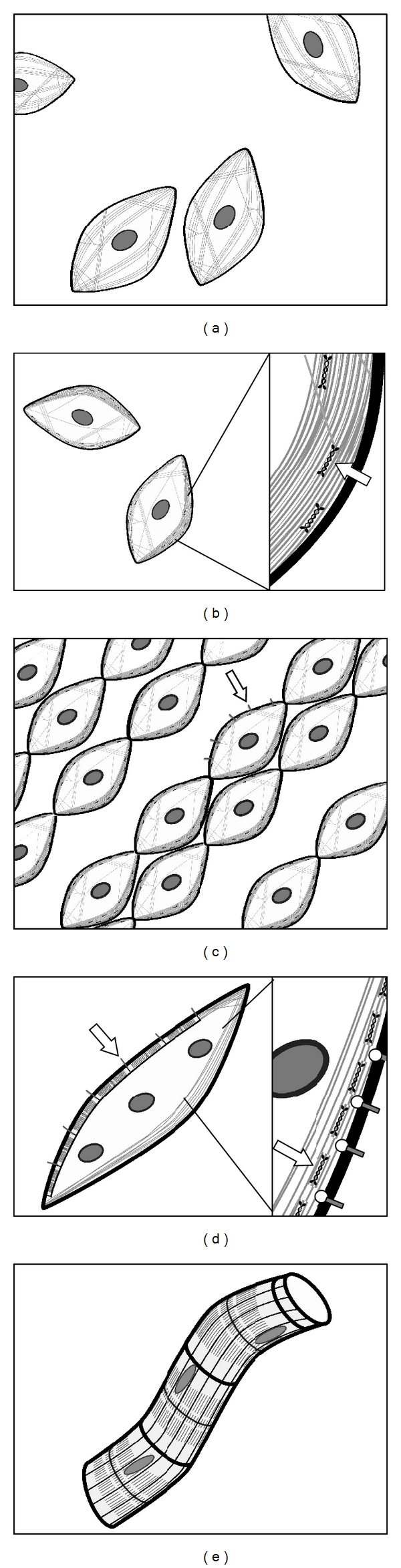
Summary of steps involved in myocyte differentiation and myofibrillogenesis, with regards to actin dynamics. (a) Proliferating myoblasts are derived from determined myotome cells and possess an unspecialized actin cytoskeleton (grey lines). (b) As differentiation begins, these cells aggregate, characterized by the formation of localized stress fibers in a cortical actin wall (insert). Contractile function of these fibers is provided by nonmuscle myosin (NMM, arrow). (c) Myoblasts align themselves concurrent to substrate attachment and the elaboration of protocostameres (arrow). The stress-fiber-like cortical actin and NMM will form the premyofibril templates for subsequent myofibril assembly. (d) Fusion occurs, resulting in the formation of multinucleated myotubes. Myofibrils begin to form at the cell periphery, centered on costamere attachment points (arrow), constructed from premyofibril templates (insert). Premyofibrils consist of alternating bands of membrane-associated *α*-actinin (circles) and NMM (arrow). (e) As the myocyte matures, additional myofibrils will fill all available space, interconnected with one another and with organelles by desmin intermediate filaments. New myoblasts will continue to fuse to the terminal ends of the myotube to create a growing myofiber. Artwork by A. Pete.

Determined myoblast progenitors from paraxial myotome and dermomyotome can first be detected by the expression of pax3 and the subsequent expression of myogenesis-regulating basic helix-loop-helix (bHLH) transcription factors of the MyoD family (reviewed in [13]). Activation of downstream myogenic gene programs by these factors results in aggregation of the proliferating myoblasts, followed by cell alignment, substrate attachment, and fusion of myoblasts into linear syncytial myotubes (Figure 1). Myofibrils first form at the cortex of the newly fused myotubes from elaborations of the actin cytoskeleton and aggregations of the Z-band protein, *α*-actinin (Figure 1(d)). These initial structures are similar to the stress fibers of motile cells and are sometimes called premyofibrils [14, 15]. These early fibrils attach to the cell surface and extracellular matrix at protein complexes called costameres (Figures 1(c) and 1(d)) [16, 17]. Additional myofibrils gradually fill the interior of the cell, anchored to one another and to myocyte organelles by desmin-rich intermediate filaments, until all available space is used, leaving narrow gaps for sarcoplasmic reticulum, mitochondria, and nuclei (Figure 1(e)). Additional myoblasts fuse to the growing ends of the myotube until the multinucleated cell becomes a mature, fully differentiated myofiber [9, 18], in which mature isoforms of myosin replace previous embryonic and neonatal isoforms as post natal development continues [19, 20].

**Figure 2:**
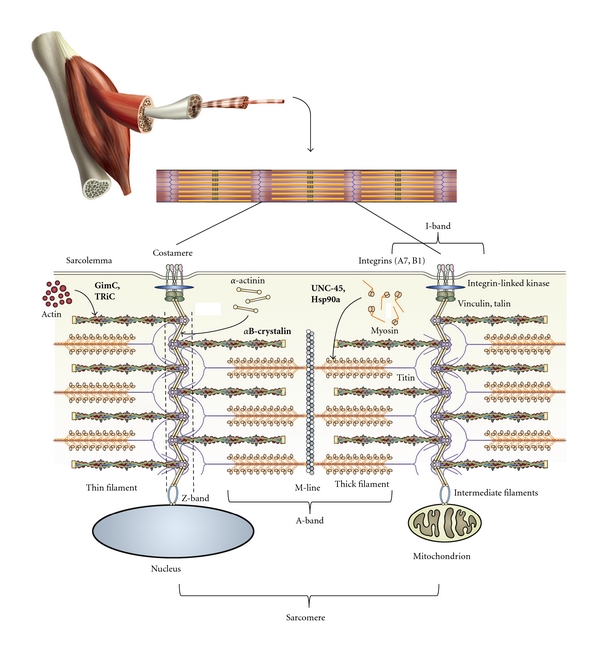
Schematic diagram of the sarcomere and costamere protein complexes of striated muscle cells. Major components of the mature sarcomere and costamere are shown, along with the cytoskeletal and motor filament systems, in context with the sarcolemma and organelles of syncytial myocytes. Known chaperone or cochaperone molecules are shown in bold, along with their substrates. Arrows indicate regions where chaperone-mediated protein folding is essential to incorporate polymeric filament proteins. Modified from Sparrow and Schock [17], additional artwork by A. Pete.

The sarcomeres of myofibrils are repeating linear protein complexes made up of overlapping protein filaments, consisting primarily of myosin, actin, titin, nebulin, and other associated proteins (Figure 2). The overlapping contractile filaments create a recognizable periodic banding pattern, consisting of regularly spaced A-bands and I-bands, which contain the myosin thick filaments and actin thin filaments, respectively, giving striated muscle tissue its characteristic appearance (reviewed in [9, 18, 21]). The thick filaments are made up of polymeric muscle myosin II, organized into hexamers consisting of 2 heavy-chain motor subunits and 4 light-chain regulatory subunits. These individual hexamers organize into higher-order bundles, with globular head domains of heavy-chain subunits jutting outwards from a central filament core, and these bundles associate tail-to-tail with the aid of myomesin, creating a bi-directional thick filament with motor heads extending in both directions. The head domains interact with the overlapping actin-rich thin filaments and perform ATP-dependent motor activity, which is controlled by calcium-mediated association of nebulin/tropomyosin with the actin thin filament. Titin fibril formation occurs in a stepwise fashion as the N-terminal peptide first associates with Z-band proteins, followed by folding and elaboration of the fibril, and finally association of the C-terminal peptide with proteins of the M-line, concurrently with thick filament assembly [22, 23]. The length of the fully assembled titin protein delineates the spacing of each sarcomere when at rest, approximately 2.5 microns in mammalian cells [24, 25]. 

### 2.2. Molecular Chaperones Are Required for Sarcomere Organization and Filament Assembly

Folding of the myosin globular head domain and higher-order assembly of sarcomere thick filaments both require the activity of muscle-specific molecular chaperones [26–28]. Myosin II fails to maintain motor function when expressed *in vitro* or in bacterial systems or is subjected to denaturation [28, 29], demonstrating the necessity for chaperone-mediated protein folding. “Chaperone” in the context of this paper refers to any factor directly responsible for protein folding or stability, regardless of a demonstrated ability to prevent protein aggregation *in vitro*. Molecules that have been implicated in assisting the proper folding of myosin II include heat-shock family proteins such as Hsp90 and Hsp70 and UNC-45 [27, 28, 30–33]. Two *α*-isoforms of the Hsp90 family (Hsp90a1 and Hsp90a2) are required for proper folding and assembly of the myosin thick filament in vertebrates, and these chaperones are specifically expressed in developing heart and skeletal muscle [34, 35], while other isoforms are more ubiquitously expressed [35, 36]. Likewise, chaperones are involved in the assembly of actin thin filaments, titin filaments, and the intermediate filaments which anchor sarcomeres laterally to each other and to organelles within the myofiber (Figure 2, reviewed in [37, 38]). These include N-RAP, which is involved with organization of *α*-actinin in the Z-band [39, 40]; GimC and TRiC, which are required for thin filament actin folding [41, 42]; the small heat-shock protein *α*B-crystalin, which is necessary for folding of the titin filament [43, 44] and for desmin folding in myocyte intermediate filaments [45, 46]. Mutations in genes encoding these chaperones or their substrates are often associated with disorganized musculature and myopathy in vertebrates [10–12, 37, 47], suggesting that the establishment of cytoskeletal scaffolds is essential for subsequent assembly of the functional motor elements of the thick filament. 

Both the actin and titin filaments are fully formed before myosin thick filament assembly is complete, leading to the long-standing hypothesis that the final spacing of the Z-bands and M-lines depends on titin assembly as a scaffold for both actin and myosin filaments [21, 48]. This giant, semirigid, modular protein attaches to both Z-band and M-line proteins, acting as a spacer to dictate the distance between the centers of the thin and thick filaments when myocytes are at rest (reviewed in [49]). Previous models of early myofibrillogenesis suggest that the association of the titin N-terminal peptide with Z-bodies is evidence that subsequent translation or assembly of the titin protein establishes the final pattern of the sarcomere; the newly added peptides recruit thick filament and M-line components as the titin protein assumes its final conformation, culminating in the formation of the M-line at the titin C-terminus [21, 49]. Several lines of evidence support this role for titin; knockout or knockdown of various titin peptides results in impaired or abolished thick filament and sarcomere organization [50–53], and deletion of the M-line region of titin allows initial myofibrillogenesis but results in subsequent disassembly of myofibrils and muscle atrophy [54, 55]. However, it is important to note that this merely demonstrates the necessity for titin during the later stages of thick filament integration with the preexisting actin thin filaments and Z-bodies, as initial pattern formation of *α*-actinin precedes that of the N-terminal titin peptide [14, 18, 56]. Further, C-terminal titin epitopes are not detectable until thick filament assembly and expression of M-line components has already begun [57, 58]. M-band-associated titin also appears to act as a signal transducer for sarcomere tension, which may indicate that the role of titin in thick filament organization is regulatory rather than physical [59]. A competing model of early myofibrillogenesis, initially proposed by Rhee et al. [14], suggests that thick-filament organization is not dependent on the presence of a preexisting titin scaffold, although titin is likely still necessary for the recruitment of thick filament components or chaperones. Rather, the nonmuscle myosin II (NMM) filaments in stress-fiber-like structures act as intermediates to the myofibril, called premyofibrils. NMM is thus replaced by muscle myosin II in a stepwise fashion, allowing thick filaments to be incorporated into the expanding actin cytoskeleton concurrently [9, 18]. Current evidence favors the premyofibril model, although the titin and premyofibril models are not necessarily mutually exclusive.

### 2.3. Early Myofibrillogenesis Involves Protein Dynamics and NMM Premyofibrils

Regardless of the precise role of titin in thick filament assembly, it is now clear that nonmuscle myosin II plays an essential part in the formation of premyofibrils, as demonstrated by a number of studies. Most notably, the alignment and fusion of cultured myoblasts requires the presence of an extensive cortical actin network and the activity of nonmuscle myosin IIA [60], indicating that the components of stress-fiber-like structures are present at the cortex of newly formed myotubes from the moment fusion occurs (Figures 1(a)–1(c)). The premyofibril model is also supported in part by the necessity for proteasome-mediated protein degradation prior to and during thick filament assembly. Inhibition of proteasome function disrupts fusion and sarcomere formation in cultured myoblasts [61, 62], and several sarcomere components and chaperones are known to interact with ubiquitin-recruiting factors [38, 63–65], indicating that constant turnover of sarcomere proteins is very likely. Indeed, studies have confirmed the rapid turnover of various sarcomere components using FRAP analysis [40, 66]. This form of protein quality-control is a common mechanism for molecular chaperones in the prevention of protein aggregation.

The first detectible pattern-forming structures within newly fused myotubes are the *α*-actinin nucleation sites, called Z-bodies, which also incorporate cortical cytoskeletal actin to form the stress-fiber-like structures mentioned previously. These structures have been referred to as I-Z-I brushes, appearing under electron microscopy as dense *α*-actinin clusters with radiating F-actin branches [67]. The titin scaffold model proposes that titin recruits Z-bodies into I-Z-I brushes, but this would imply that Z-bodies are discrete and randomly distributed, which does not appear to be the case. Rather, as the premyofibril model proposes, these membrane-associated clusters of *α*-actinin form regular periodic patterns, alternating with bands of NMM-II within the actin premyofibrils [68–70]. These bands form a minisarcomere with a smaller period (0.3–1.5 microns) than the mature sarcomeres of striated muscle, but which appears to act as a fully functional contractile system in early myotubes. The repeat period of minisarcomeres was observed to grow over time [69], as thick filament muscle myosin II replaced premyofibril NMM-II, concurrent with titin filament elaboration. Although the N-terminal titin peptide is present in Z-bodies and I-Z-I brushes, C-terminal titin epitopes are not detectable until later in myofibrillogenesis [57, 58].

## 3. The Roles of Nonmuscle Myosin and Cell Attachment during Myofibrillogenesis

### 3.1. Myosin II Motor Function during Myofibrillogenesis

The requirement for myosin motor function during myofibrillogenesis has been recognized for some time since inhibition of myosin heavy chain (MHC) motor activity using pharmacological inhibitors suppresses the formation of organized myofibrils in cultured myoblasts [71, 72]. Further, pharmacological inhibition of contractile signals by calcium channel blockers causes myofibril disassembly and loss of cell-substrate attachment in mature cultured myocytes [73, 74], indicating a continued need for tension across the cell to maintain sarcomere integrity. Inhibition of myosin contractility also severely reduces sarcomeric actin dynamics in developing cardiomyocytes [75], and even the loss of contractile regulation by a calcium-signal-transducing myosin-light-chain kinase (MLCK) inhibits thick-filament assembly during myofibrillogenesis [76–78]. Further, electrical stimulation of contractility can overcome the effects of calcium signal blocking or even accelerate myofibrillogenesis in untreated cells [79]. None of these methods of myosin II contractile inhibition are specific only to muscle-myosin II; nonmuscle myosin activity is likely affected equally by myosin motor inhibitors and inhibitors of calcium signalling. Indeed, specific inhibition of a nonmuscle myosin light chain kinase results in reversible inhibition of myofibril assembly [78], and specific depletion of NMM mRNA from undifferentiated myoblasts in culture impairs the polymerization of cortical actin, blocking myoblast fusion and subsequent myofibrillogenesis [60, 80]. Taken together, these data imply that the necessity for myosin motor function during muscle development significantly precedes incorporation of muscle MHC II into the thick filament.

Criticisms of the premyofibril model generally arise from the observation that the nonmuscle myosin isoform generally associated with premyofibrils is NMM-IIB, while NMM-IIA is the isoform most often associated with stress fibers in nonmuscle cells [21, 81]. Since NMM-IIA is specifically necessary for skeletal myoblast fusion [80], while NMM-IIB appears to be the primary isoform in pre-myofibils of cardiomyocytes, it seems likely that different isoforms possess specialized roles in different processes, and knockdown studies in cultured myoblasts have supported this idea [80]. Further, knockout mice lacking NMM-IIB, which do not survive past birth and have malformed hearts, still contain a significant proportion of normal cardiac myofibrils, indicating that not all myofibril formation depends on NMM-IIB [82, 83]. It is important to note, however, that these embryonic hearts also contain increased levels of the NMM-IIA isoform, which may play a redundant role during myofibrillogenesis; indeed, co-knockdown of NMM-IIA with -IIB demonstrates a more severe phenotype, although overexpression of IIA alone cannot rescue the IIB knockout phenotype [83]. The newly discovered NMM-IIC, of which little is known, may also have redundancy with IIA and IIB [84]. All three isoforms of NMM-II are normally detectable in developing myocytes, and both NMM-IIA and -IIB are localized to the cortical actin wall after fusion [84, 85]. Redundancy between myosin isoforms is not unprecedented; in *C. elegans*, one MHC isoform has the ability to fully compensate for the loss of another during thick filament assembly, as we will discuss below [86–88]. The possibility of redundancy between the NMM isoforms thus remains a strong defence of the premyofibril model.

### 3.2. Integrins and the Role of Cell-Matrix Attachment

Myofibrillogenesis requires rigid substrate attachment with the extracellular matrix [89–91], supporting the notion that tension across the developing myocytes must be generated by myosin motor activity for myofibrillogenesis to occur. Not only does the molecular composition [92–94] and shape [95–97] of the substrate affect the ability of myoblasts to differentiate and align themselves prior to fusion, but even mature myocytes cultured in suspension cannot maintain organized myofibrils [90, 98]. Mechanical force and tension is important for regulating the formation of complex protein structures in nonmuscle cells; the formation of focal adhesion complexes depends on both substrate rigidity [99] and myosin motor function [100], and the application of external force across nonmuscle cells induces focal adhesion growth and elaboration [101]. Attachment of myocytes to their surrounding extracellular matrix (ECM) occurs in a similar fashion, via junction complexes that contain many of the same proteins as focal adhesions in nonmuscle cells. Sarcomeres nearest to the sarcolemma interact with ECM-bound integrin dimers at protein complexes called costameres [16, 17]. These complexes are localized to the lateral edges of the Z-band, where two sarcomeres are joined at the plus ends of the actin thin filaments (Figure 2), and contain many of the same components as nonmuscle focal adhesions. Indeed, it has been directly demonstrated that force generation between cultured myocytes and their substrates is mediated through costamere junctions [102]. Furthermore, inhibition of actin/myosin contractility in cultured myocytes causes disassembly of costamere protein complexes concurrent to the disassembly of myofibrils [74], and stimulation of contractility induces or reestablishes costamere organization concurrent to myofibril organization [79], indicating that costamere and myofibril assembly are closely linked. Myofibril formation can be inhibited merely by treating myoblast cultures with an RGD peptide, which antagonizes the matrix-binding region of integrin dimers, even when myosin motor activity is stimulated concurrently [79], demonstrating an essential role for integrin-matrix interaction during myofibrillogenesis.

The first sign of periodic protein alignment similar to that seen in mature sarcomeres occurs during premyofibril formation at the myocyte cortex, where *α*-actinin colocalizes with integrins, vinculin, and talin during the formation of costameres [56, 79]. Integrin-ECM adhesion sites may serve as points of nucleation for *α*-actinin, and the subsequent formation of Z-bodies, indicating that the first step in the higher-order assembly of the sarcomere is cell-substrate attachment (reviewed in [17]). Indeed, integrins and integrin ligands such as perlecan and laminin are indispensable for myofibril and sarcomere formation [103–105]. Muscle-specific deficiencies in integrin adhesion result in myopathies characterized by sarcomere disorganization and dissociation of sarcomeres from the sarcolemma [105, 106]. Additionally, genetic analysis in *C. elegans* and *Drosophila* has shown that integrin requirements are genetically upstream of other essential sarcomeric proteins such as titin [107, 108]. Muscle cell precursors in the* Drosophila* integrin mutant *mys* differentiate into myoblasts and fuse into multinucleated myotubes but form no premyofibril structures [109], further supporting the theory that costamere formation precedes the alignment of Z-bodies along premyofibrils to form the Z-band of mature sarcomeres. Integrin adhesion sites are thought to be sites of actin nucleation, mediated through Rho-GTPase signaling [110, 111]. Consistent with this, integrin attachment complexes can induce actin nucleation *in vitro*, producing filaments similar to the orientation of actin filaments found in premyofibrils [112].

**Figure 3:**
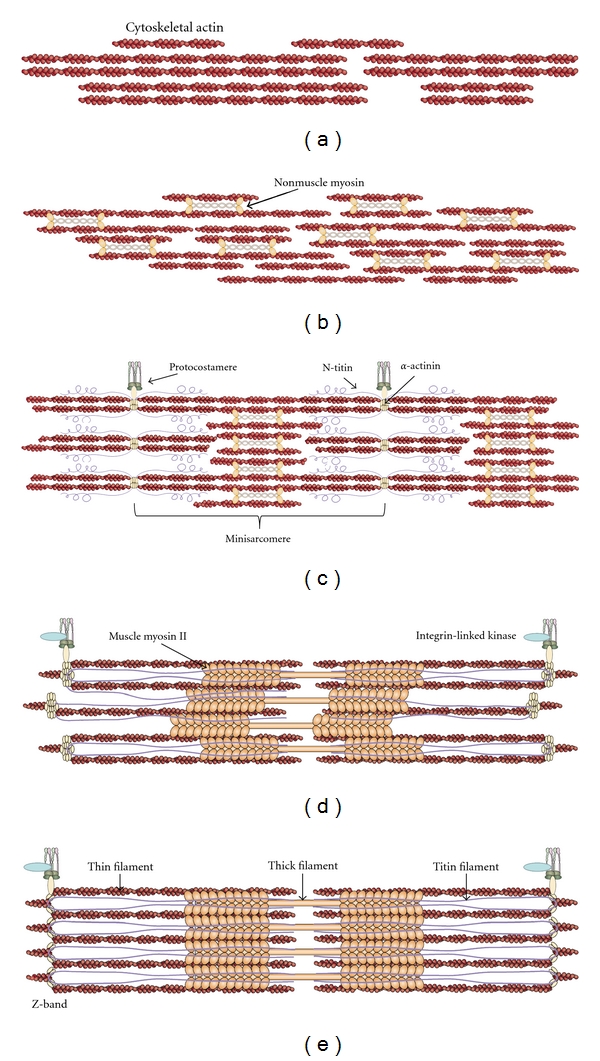
Synthesis of the premyofibril model with the roles of nonmuscle myosin (NMM) in early differentiating myoblasts. Schematic representation of the molecular events leading from cytoskeletal actin to mature myofibrils. (a) Elaboration of the actin cytoskeleton in proliferating myoblasts leads to the formation of a cortical actin wall. (b) Stress-fiber-like structures in the cortical actin wall contain associated NMM-II, which allows for alignment and fusion of myoblasts. (c) Alignment and fusion are concurrent with early costamere formation, resulting in the anchorage of premyofibrils to the extracellular matrix. These sites serve as nucleation points, resulting in the formation of minisarcomeres with alternating bands of *α*-actinin and NMM. Incorporation of N-terminal titin occurs at this point as well. (d) Folding and lengthening of titin is concurrent with the organization of *α*-actinin into the Z-band and the incorporation of muscle MHC-II into the thick filament, displacing NMM and widening sarcomeres. (e) M-line proteins associate with MHC-II and C-terminal titin, creating the final banding pattern of mature myofibrils. Modified from Sparrow and Schock [17].

Integrins are also proposed to require stabilization by molecular chaperones, and interactions have been demonstrated between *β*1 integrin, integrin-linked kinase (ILK), and Hsp90 [113, 114]. Stabilization of ILK is required for the assembly of costameres; however, the specific role played by chaperones in this process is not understood, and few of the proteins involved have been characterized [17, 79, 115]. Protein aggregates are often found near Z-bands in myopathies associated with sarcomeric scaffolding proteins, indicating the need for regulation of protein assembly and stability by chaperones in the vicinity of costameres [15, 38]. A synthesis of the premyofibril model with the integrin/costamere model would, therefore, propose the following order of events (outlined in Figure 3): first, the actin cytoskeleton of proliferating myoblasts is modified to create the cortical actin wall, which associates with NMM prior to aggregation and fusion (Figures 3(a) and 3(b)). Second, formation of integrin-ECM attachments occur, concurrent with myoblast fusion. Next, the integrin attachment complexes serve as nucleation sites for *α*-actinin, vinculin, and talin, creating regularly-spaced, membrane-associated Z-bodies. Further actin polymerization mediated by formins occurs at these sites as well, resulting in the creation of the minisarcomeres that are often called I-Z-I brushes; these are characterized by alternating bands of *α*-actinin and nonmuscle myosin, with associated N-terminal titin epitopes (Figure 3(c)). These premyofibrils grow and move costameres farther apart, as titin is incorporated into the protosarcomeres, concurrent with the replacement of NMM with muscle myosin II (Figure 3(d)). This eventually results in the final spacing of thin and thick filaments with a Z-band period of 2.5 microns (Figure 3(e)). Specific chaperone activities are necessary throughout this process, including the stabilization of ILK and integrin attachment complexes, the nucleation and organization of *α*-actinin into Z-bodies and mature Z-bands, the polymerization of actin, and the folding and assembly of muscle myosin to form the mature thick filaments (listed in Figure 2).

## 4. UNC-45 and Myosin Assembly during Myogenesis

### 4.1. Current Models of UNC-45 Function during Myofibrillogenesis

**Figure 4:**
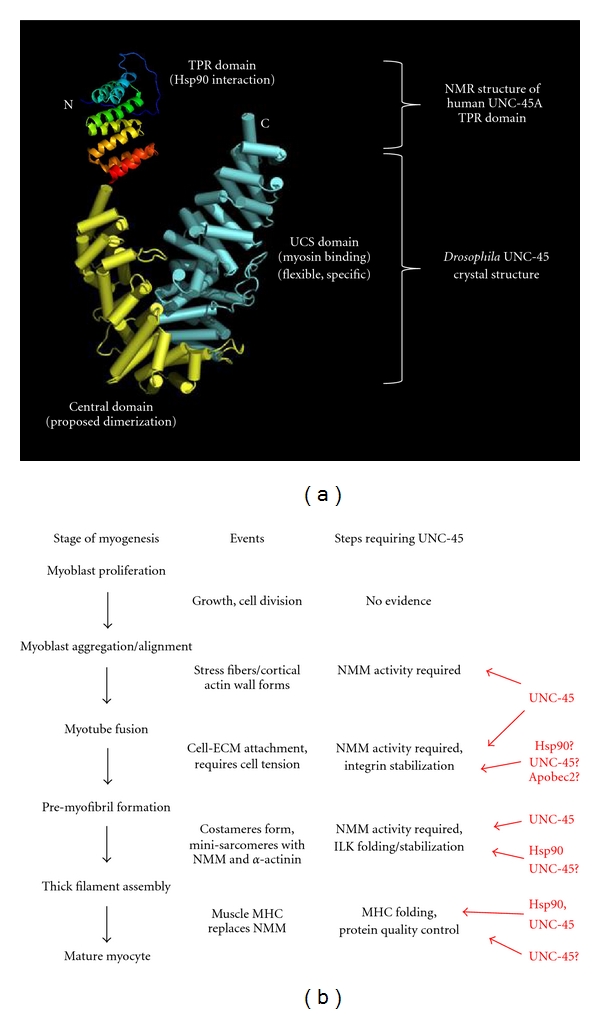
Proposed model of roles for Unc45b/nonmuscle myosin during early myofibrillogenesis. (a) Merged protein model of Unc-45b from the X-ray crystal structure of *Drosophila* UNC-45 and the solved NMR structure of the human Unc45a TPR domain (protein database ID 2DBA), showing the known active domains. Proposed functions of each section of the protein are indicated. Modified from Lee et al. [139]. (b) Flowchart of myofibrillogenesis, listing the stages where there is significant evidence to hypothesize the involvement of UNC-45. Events that likely involve UNC-45 chaperone function at each stage of myogenesis are noted, and probable cofactors for UNC-45 are indicated in red.

UNC-45 is a myosin-binding protein, initially identified in *C. elegans* through mutations affecting the proper assembly and function of thick filaments in body wall muscle [116, 117]. Invertebrates have a single UNC-45 isoform, while two isoforms are found in vertebrates, called Unc45a and Unc45b [118]; Unc45a is expressed ubiquitously, and mutations in zebrafish affect heart development but not striated muscle formation [119]. By contrast, Unc45b is expressed specifically in striated muscle, in a pattern virtually indistinguishable from that of Hsp90a [35, 36, 120]. *C. elegans* UNC-45 interacts with Hsp90 family members through an amino-terminal tetratricopeptide repeat (TPR) domain [31, 117], and with muscle myosin heavy-chain B (MHC-B) through a ~400 residue UNC-45/Cro1/She4p (UCS) domain that is conserved with fungal homologues [116, 121] (Figure 4(a)). UNC-45/Unc45b is proposed to act as a cochaperone with Hsp90a, for the folding and assembly of MHC-II in striated muscle. The specific requirement for Hsp90a chaperone activity in myofibrillogenesis is well documented [34, 122–124], and the mechanisms of Hsp90 ATPase-dependent protein folding are becoming understood (reviewed in [125, 126]). However, less is known about the nature of requirements for tissue-specific cofactors like Unc45b. Reducing Unc45b function in zebrafish or *Xenopus* results in loss of thick filament assembly and disorganization of sarcomeres [35, 120, 127]; indeed, the phenotype of zebrafish Unc45b mutants (*steif*) [35] is very similar to that of Hsp90a1 mutants (*sloth*) [124], characterized by poorly organized sarcomeres, loss of thick filament assembly, heart dysfunction, pericardial edema, and paralysis.

Hsp90a and Unc45b transcripts are both upregulated in Unc45b/*steif* mutant embryos [35], and Unc45b expression is increased in response to protein stress in a similar fashion to Hsp90a [128]. This suggests that Hsp90a and Unc45b may also be coregulated during zebrafish muscle development or following protein stress. Additionally, the subcellular localization and dynamics of Unc45b and Hsp90a during sarcomere assembly and protein stress has been examined in whole zebrafish embryos expressing fusion proteins [128, 129]. Rather than localizing to the myosin thick filament, the Unc45b/Hsp90a complex was found to localize to the Z-bands and myosepta of developing myocytes, subsequently shifting to the thick filament A-band in response to protein stress. It was proposed that the *α*-actinin Z-band may act as a reservoir for stress-response chaperones; however, this does not explain localization to the myoseptum, which contains a relatively different protein complement. Alternatively, the Z-band localization of Unc45b/Hsp90a may represent association with dynamic cell-surface costamere junctions, where they may act to stabilize ILK or other costamere components [114].

### 4.2. UNC-45 May Exhibit Direct Myosin Chaperone Activity

UNC-45 has been demonstrated to have chaperone activity *in vitro* [31, 130], and Unc45b-Hsp90a complexes are necessary for the folding of the MHC motor domain in vertebrates [33, 122]. Vertebrate Hsp90a1 and Hsp90a2 are associated only with partially folded intermediate forms of myosin [31], supporting the hypothesis that cofactors such as UNC-45 are necessary for the proper folding and assembly of sarcomeric myosins during myogenesis. *Drosophila* UNC-45 is able to reduce heat-induced myosin aggregation *in vitro*, without the addition of any other chaperone or co-factor [130]. However, vertebrate Unc45b has little effect on MHC folding without the addition of Hsp90a [33], and it has not yet been shown that UNC-45 directly mediates protein folding *in vivo*. The primary role of Unc45b in vertebrates may be to act as an adaptor molecule, stabilizing the interactions between Hsp90a and MHC through the TPR and UCS domains, respectively. However, protein aggregation may also be prevented somewhat indirectly by chaperones that target misfolded proteins for degradation. Such a role has been suggested for UNC-45 in *C. elegans*, following studies that show interaction between UNC-45 and the CHN-1/UFD-2 ubiquitylation complex. [63]. Overexpression of UNC-45 in *C. elegans *results in ubiquitin/proteasome-mediated myosin degradation, indicating that UNC-45 may act to prevent the accumulation of misfolded myosins [64], another common function of molecular chaperones. In humans, the ubiquitin-selective chaperone p97, known to cause hereditary inclusion-body myopathy in mutants, regulates UNC45B degradation [38, 131]. *UNC45B* has, therefore, been proposed as a candidate locus for additional cardiomyopathies [132]. This is consistent with the observation that overexpression of vertebrate Unc45b in zebrafish results in a similar phenotype to knockdown embryos, with disorganized sarcomeres and loss of thick filament assembly [133]. Despite these reports, however, it remains unclear whether chaperone functions of UNC-45 are directly accomplished or mediated entirely through its association with Hsp90a.

### 4.3. UNC-45 Shows Myosin Isotype Specificity during Development

Unlike Hsp90 family members, UNC-45 in *C. elegans *is not a general chaperone but rather shows striking isotype specificity. Thick filaments in *C. elegans* are assembled from two different muscle myosin heavy chains, MHC-A and -B, which are asymmetrically arranged within the filaments. The minor isoform (MHC-A) is found only in the central 2 *μ*m of the filament, while MHC-B is found along the majority of the lateral arms [134]. In the region corresponding to the M line of vertebrate sarcomeres, where only MHC-A is found in *C. elegans*, the myosin molecules adopt an antiparallel alignment. It may be that the MHC-A tail domain more readily accommodates the antiparallel arrangement or more readily assembles on the paramyosin core to initiate thick filament organization [86, 87], while MHC-B may be a more efficient motor protein. If MHC-B is removed by mutation (*unc-54 *null mutants), thick filaments can still be induced to form entirely from MHC-A [88]. In these animals, UNC-45 no longer localizes to the thick filaments, and missense mutations in UNC-45 have no effect on thick filament assembly [116]. Thus, MHC-A does not seem to require UNC-45 activity despite the fact that the two myosins are 65% identical at the sequence level, and there are no obvious regions of difference in the sequence alignment that would explain this difference in function [86]. There is also no reciprocal rescue; null mutations in MHC-A are embryonic lethal whether or not MHC-B is overexpressed [135].

In addition to MHC-B, UNC-45 in *C. elegans* interacts with the nonmuscle myosin, NMY-2 [136]. UNC-45 colocalizes with NMY-2 during cytokinesis of the early embryo, where it seems to regulate cell contractility [136], and this colocalization has also been shown in the early blastoderm of 2-hour-old *Drosophila* embryos [137]. In both species, UNC-45 must be maternally contributed to the oocyte, appearing well before the midblastula transition. The nematode maternal gene product can partially ameliorate the muscle phenotype in UNC-45 mutants, making it unique among muscle genes in *C. elegans* [117]. In the nematode gonad, there are two type II nonmuscle myosins, NMY-1 and NMY-2. However, only NMY-2 seems to require UNC-45 activity [T. Kachur and D. Pilgrim, in preparation]. Furthermore, the interaction of UNC-45 with NMY-2 seems to happen at a late stage of myosin assembly, as NMY-2 and actin can fully assemble into identifiable stress fibers, but these structures cannot contract [138], implying that the role of UNC-45 in this context is related to motor function rather than filament assembly. Thus, rather than acting as a general myosin chaperone, UNC-45 is required for isoform-specific myosin activity. Additionally, crystal analysis of *Drosophila* UNC-45 (dUNC-45) suggests that its myosin-specific chaperone activity can be attributed to the flexibility of the elastic UCS domain [139]. This is in keeping with recent structural studies that suggest a role for the yeast UCS homologue, She4p, in limiting step size during myosin motor action [140] (Figure 4(a)). Moreover, dUNC-45 expression colocalizes with both nonmuscle myosin and PS2 integrin at Z-bands during sarcomere formation in third-instar larvae [137, 141]. It has thus been suggested that Z-band nonmuscle myosin in *Drosophila* plays a role in cross-linking actin to provide mechanical stability to the sarcomere, and it is possible that dUNC-45 helps to mediate this function.

## 5. Unc45b Seems to Play an Earlier Role in Myofibrillogenesis

### 5.1. UNC-45 Interaction with Invertebrate Nonmuscle Myosins Implies an Earlier Role in Myogenesis

While the phenotype of temperature-sensitive *UNC-45 *mutant alleles is suppressed when the thick filaments are assembled from MHC-A as described above, UNC-45 null mutant alleles in this background are still embryonic lethal. One possibility is that small quantities of UNC-45 protein are necessary for reasons unrelated to thick filament assembly. The terminal phenotype of these null alleles, known as Pat (paralyzed and arrested at two-fold stage), is similar to the Pat phenotype of mutations in genes required for the earliest stages of myoblast differentiation and myofilament assembly [117, 142], including ECM components and the *α* and *β*-subunits of integrin (*pat-2, -3*) [143]. Given the interaction of UNC-45 with NMY-2 [136, 138] and the myofibrillogenesis models that propose integrin-ECM attachment as the first step in NMM-dependent premyofibril assembly [9, 17], this phenotype suggests a hypothesis that UNC-45 plays a much earlier role in myofibrillogenesis than previously thought, one independent of muscle myosin MHC, but perhaps requiring other myosins such as NMM. In vertebrates, Unc45b has not been rigorously tested for a role in early embryonic cytokinesis or interactions with nonmuscle myosins. Rather, it has widely been assumed that the more ubiquitous Unc45a isoform performs these general cell functions, while Unc45b acts in a muscle-specific fashion, consistent with their patterns of expression. It has also been assumed that the muscle-specific activity of Unc45b is entirely related to its interactions with Hsp90a and muscle MHC, and two-hybrid screens have not yet indicated NMM as a substrate for Unc45b binding. Given that protein-interaction screens with complex-forming chaperone molecules are notoriously difficult to accomplish in yeast, as multiple cofactors are often necessary to stabilize interactions, this hypothesis should not be excluded as a possibility. Multiple lines of evidence suggest that Unc45b performs a muscle-specific function during early myofibrillogenesis, prior to thick filament assembly.

### 5.2. Evidence for an Earlier Myofibrillogenesis Role from Vertebrate Unc45b

In zebrafish, Unc45b mRNA can be detected in the paraxial mesoderm of developing zebrafish embryos prior to somite formation or myoblast differentiation [120, 144], during the earliest stages of myogenesis. The onset of Unc45b expression in cultured zebrafish blastomeres undergoing myogenic differentiation is concurrent with that of the early myoblast marker, MyoD, well before the expression of muscle MHC [145]. After alignment and fusion of myoblasts, Unc45b protein localizes to the developing Z-bands and the myosepta (where the myocyte termini of adjacent somites meet), which are regions of cell-ECM attachment, rather than to the myosin thick filament as would be expected for a muscle myosin chaperone [128, 129]. Hsp90 interacts with and stabilizes ILK, a major component of signalling pathways at the costamere [114], and Unc45b may also interact with some element of the costamere attachment complex. However, lateral attachment between myofibers in Unc45b mutants is reduced, with large gaps appearing between mature myocytes in 3- to 5-day-old larvae, a phenotype which has not been reported in Hsp90a mutants [35, 129]. This is consistent with a role for Unc45b in the formation or maintenance of costamere anchoring complexes, separate from the role of Hsp90 in stabilizing integrins and ILK. The Z-body nucleation complexes which form downstream of integrin attachment at costamers contain *α*-actinin, nonmuscle myosin, and the PDZ-LIM-domain protein ZASP [146, 147]. The ZASP complex has been suggested to act as a sarcomere-stabilizing tension sensor, and the absence of nonmuscle myosin from this complex reduces thick filament organization [147].

An Unc45b binding partner, Apobec2, appears to co-localize to costamere attachment regions and interacts with Unc45b but not with Hsp90a in pull-down assays [129]. Apobec2 is expressed in differentiating myocytes, and knockout mice are characterized by low muscle mass and nonlethal myopathy [148], while depletion of Apobec2 in zebrafish results in a dystrophic myopathy with deficiencies in lateral myocyte attachment and organization of the myosepta [129]. These embryos display gaps between myocytes similar to the phenotype observed in Unc45b mutants [35, 129]. The zebrafish *hsp90a2* mutation, *sloth*, disrupts the late stages of thick filament integration, but not premyofibril formation or the organization of the Z-band, while the Z-band in Unc45b mutants is highly disorganized, suggesting that earlier Unc45b activity is required for Z-band organization in a non-Hsp90-dependant manner [35, 124]. Further, it has been shown in *C. elegans* that the UNC-45 mutant phenotype can be partially ameliorated by the expression of truncated UNC-45 lacking the TPR domain [149], demonstrating that at least some of UNC-45 function occurs independent of Hsp90a. None of these studies have examined Unc45b localization during early myofibrillogenesis, although the initial formation of costameres and the maintenance of the mature attachment complex may both involve similar chaperone activity. Apobec2 is expressed in differentiating myoblasts prior to fusion [148], and so it will be interesting to see at what point Apobec2 and Unc45b co-localize to the myoseptum during development.

Mutations in the *Xenopus *Unc45b homologue *dicky ticker* result in delayed formation of Z-bodies and reduced polymerization of *α*-actinin in developing somites [127], indicating deficiencies in the early cytoskeletal organization at costameres. Given the established theory that *α*-actinin nucleation occurs at protocostameres [17], concurrent with premyofibril organization from cytoskeletal actin and NMM [9], this also supports an early premyofibril role for Unc45b. As UNC-45 plays a role in mediating *C. elegans* nonmuscle myosin function during oocyte cytokinesis and later gonad maturation [138], Unc45b may play a similar role in mediating NMM function in vertebrates during the organization of premyofibrils and costamere attachment to the ECM. This would explain the deficiencies of myocyte attachment in zebrafish mutants and the early onset of Unc45b expression and provide a mechanism by which NMM function during myoblast fusion and premyofibril organization is coupled to the same chaperone complexes that later mediate the folding and assembly of thick filament myosin. In particular, the lack of contractile function in NMY-2 stress fibers formed in *C. elegans* UNC-45 mutant embryos, which otherwise appear normal [138], indicates that UNC-45 mediates function rather than folding of nonmuscle myosins. This is also consistent with the proposed functions of UCS protein homologues in fungal systems [140]. Given the myofibrillogenesis defects following inhibition or depletion of NMM-IIA and -IIB in cultured myoblasts [60, 80] and *Drosophila* larvae [147], it is possible that Unc45b may chaperone NMM function during the premyofibrillogenesis of striated muscle, and that only the later stages of MHC thick filament assembly are mediated by Hsp90 family members.

This is the background to the model shown in Figure 4(b), and the hypothesis that UNC-45 may be involved at a number of different points during early myofibrillogenesis, prior to muscle MHC folding and thick filament assembly. These include interactions with NMM-IIA or -IIB to regulate their function during myoblast aggregation, fusion and premyofibril formation; the stabilization of integrin attachment points at protocostameres in a complex with Apobec2; regulation of signalling at costameres by Hsp90-mediated folding of ILK; or the maintenance of costamere-substrate tension via functional regulation of NMM-IIB. It is unclear whether the mechanisms of Unc45b activity involve direct chaperone folding, stabilization of substrates by targeted degradation of misfolded protein, or regulation of NMM motor function; indeed, all three mechanisms may play a part.

## 6. Conclusions

### 6.1. Integrin Junction Sites and Costamere Formation Are the First Steps in Sarcomere Organization

The organization of sarcomeric protein complexes during early myoblast differentiation remains poorly understood, and although many factors involved in the process have been identified, the specific order of events that leads from proliferating myoblasts to differentiated myotubes with mature myofibrils is not clear. However, integrin-ECM interactions and subsequent costamere formation are implicated as the initial steps in establishing the periodicity of regularly-spaced sarcomeres. Integrin adhesion precedes *α*-actinin nucleation and the spacing of Z-bodies into proto-sarcomeric I-Z-I brushes, and costamere complexes at integrin junctions are likely the initial sites of *α*-actinin nucleation. Loss of integrin function or blocking of integrin substrate binding results in loss of subsequent sarcomere organization in cultured myotubes, and this process is dependent on myosin motor function and cell tension, despite the lack of muscle myosin II expression at these early stages of differentiation. Further, the presence of the cortical actin wall and the necessity for NMM function during fusion of myoblasts implies that the earliest steps in premyofibril formation occurs at the extreme outer periphery of the cell, where cell-matrix interactions are occurring, and that cell tension is initially mediated by NMM. This is in keeping with observations that *α*-actinin nucleation is preceded by early costamere assembly, and the dependence of focal adhesion complexes on NMM function in nonmuscle cells.

### 6.2. Unc45b Seems to Play a Role in Myofibrillogenesis prior to Thick Filament Assembly

Due to the early, pre-somitigenesis expression of Unc45b in zebrafish, which significantly precedes expression of muscle MHC II, it seems logical to presume a functional role for this protein that is separate from thick filament assembly. In particular, the loss of lateral attachment between myofibers in Unc45b/*steif* mutant embryos, the colocalization of Unc45b and Apobec-2 to myosepta and the Z-band of developing myocytes, and the reduction of *α*-actinin nucleation in *Xenopus* mutants would seem to indicate a possible role in early organization of costameres and premyofibrils. Nonmuscle myosin has a proposed key role in early vertebrate myoblast fusion and patterning of the premyofibrils, and the functional dependence of NMY-2 upon UNC-45 in *C. elegans *has been clearly established. This leads to a model whereby UNC-45 has an essential role in the earliest stages of myocyte patterning and myofibrillogenesis separate from its role in thick filament assembly, mediating either the folding and function of nonmuscle myosin in stress-fiber-like premyofibrils, and/or acting in a chaperone complex with Apobec2 at costamere junctions sites. Mediation of ILK folding by chaperones has also been demonstrated, although not all of the molecules involved have yet been characterized. These possibilities are not mutually exclusive, as costamere assembly and stabilization has been shown to depend on myosin motor function and the maintenance of tension across the cell, and UNC-45 may be involved in stabilizing both nonmuscle myosins and costamere components.

This model, therefore, predicts that Unc45b interacts with NMM in vertebrates, and colocalizes with NMM or costamere components during early premyofibrillogenesis and the formation of costamere attachments. Further, it predicts that increased costamere and myofibril growth following stimulation of myosin motor activity will be inhibited in Unc45b mutant cells. It will be important to determine the molecular consequences of inhibiting NMM motor function in otherwise normal cells, to attempt to recreate the effects of Unc45b mutation in early myofibrillogenesis. Teasing out the different roles played by this multifunction chaperone at different stages of myogenesis will represent an intriguing challenge for researchers interested in the molecular events underlying early muscle development and myofibril formation.
